# Characteristics of patients meeting the new definition of pre-capillary pulmonary hypertension (Nice 2018) in a single Japanese pulmonary hypertension center

**DOI:** 10.1186/s12890-021-01623-2

**Published:** 2021-08-09

**Authors:** Keiko Yamamoto, Nobuhiro Tanabe, Yukiko Takahashi, Akira Naito, Ayumi Sekine, Rika Suda, Takayuki Jujo Sanada, Toshihiko Sugiura, Ayako Shigeta, Seiichiro Sakao, Koichiro Tatsumi

**Affiliations:** 1 Department of Respirology, Graduate School of Medicine, Chiba University, 1-8-1 Inohana Chuou-ku, Chiba, 260-8670 Japan; 2 Groupe de Recherche en Hypertension Artérielle Pulmonaire, Institut Universitaire de Cardiologie Et de Pneumologie de Québec - Université Laval, Quebec, Canada; 3 Saiseikai Narashino Hospital, Narashino-shi, Izumi-cho, Chiba, 275-8580 Japan; 4 Vrije Universiteit Medische Centrum, De Boelelaan 1117, 1118, 1081 HV Amsterdam, The Netherlands

**Keywords:** Pulmonary arterial hypertension, World Symposium on Pulmonary Hypertension (WSPH) 2018, Pulmonary artery wedge pressure, Pulmonary vascular resistance

## Abstract

**Background:**

The 6th World Symposium on Pulmonary Hypertension (Nice 2018) proposed a new definition of pre-capillary pulmonary hypertension (PH) as a condition with mean pulmonary artery pressure (mPAP) > 20 mmHg, pulmonary artery wedge pressure  ≤ 15 mmHg, and pulmonary vascular resistance (PVR) ≥ 3 Wood units (WU). The characteristics and prognosis of patients with pre-capillary PH, according to this new definition, is unclear. Therefore, we determined the characteristics and survival of patients with borderline pre-capillary PH.

**Methods:**

We retrospectively enrolled 683 patients who underwent their first right heart catheterization at Chiba University, Japan. Among them, 489 patients met the pre-capillary PH requirement with mPAP ≥ 25 mmHg (conventional pre-capillary PH group), while 22 patients met the borderline pre-capillary PH criteria (borderline pre-capillary PH group). Additionally, 16 patients with a mean PAP of 20–25 and PVR of 2–3 WU were also examined.

**Results:**

The borderline pre-capillary PH group comprised 4.3% of the total patients with pre-capillary PH, and the majority was in Group 3 (40.9%) or 4 (45.5%). The survival of the borderline pre-capillary PH group tended to be better than that of the conventional pre-capillary PH group. The prognosis of Group3 PH was the worst among the patients with borderline precapillary PH. There was no significant difference in survival between the borderline pre-capillary PH group with PVR ≥ 3 WU and that with PVR of 2–3 2WU, although none of the patients in the latter group died due to right heart failure.

**Conclusions:**

This is the first study conducted in a PH center in an Asian country to reveal the characteristics of patients with pre-capillary PH, according to the Nice 2018 definition. They comprised 4.3% of the total population with pre-capillary PH, and the majority of the pre-capillary PH cases were in either Group3 or 4. The prognosis may be affected by the patients’ underlying diseases. Further prospective studies are needed to determine whether the new definition, including the PVR cut-off, is beneficial in clinical practice.

## Background

The World Symposium on Pulmonary Hypertension (WSPH) considered the scientific and clinical knowledge concerning pulmonary hypertension (PH) and proposed a new definition for PH and new treatment strategies. The general purpose of the clinical classification of PH is to categorize clinical conditions associated with PH based on similar pathophysiological mechanisms, clinical presentation, hemodynamic characteristics, and therapeutic management. A comprehensive and simplified updated version of the clinical classification of PH was presented in Nice 2018 [1]. To date, since the first WSPH in 1973, PH has been arbitrarily defined as mean pulmonary arterial pressure (mPAP) ≥ 25 mmHg at rest. However, recent data obtained for patients undergoing right heart catheterization (RHC) have shown that normal mPAP was 14.0 ± 3.3 mmHg in healthy subjects, and two standard deviations above this mean value would suggest that mPAP > 20 mmHg is above the upper normal limit [1, 2]. In addition, the mPAP value is inadequate to define pulmonary vascular disease since this value can be affected by the cardiac output (CO) or pulmonary arterial wedge pressure (PAWP). Considering these aspects, the task force of the 6th WSPH in 2018 proposed that if all three criteria are met, namely, mPAP > 20 mmHg, PAWP ≤ 15 mmHg, and pulmonary vascular resistance (PVR) ≥ 3 Wood units (WU), then the new definition of pre-capillary PH would be satisfied [1]. A PAWP > 15 mmHg is defined as post-capillary PH, which is considered as isolated PH when PVR < 3 WU, and combined pre- and post-capillary PH when PVR ≥ 3WU. In terms of pulmonary vascular disease, the reliability and validity of this new PH definition have not been defined. Further, the characteristics and survival of patients with pre-capillary PH, diagnosed according to this new definition, are unclear. Moreover, a recent study has revealed that patients with PVR ≥ 2WU and scleroderma had a significantly poor prognosis [3].Therefore, in our cohort of patients with PH, we determined the characteristics and prognosis of patients with pre-capillary PH who had pulmonary vascular disease with a modest elevation in mPAP (borderline pre-capillary PH group). This study was conducted in a Japanese PH center that is associated with respiratory medicine and is one of the high-volume pulmonary endarterectomy (PEA) centers in Japan.

## Methods

### Ethics approval and consent to participate

Patient identity was concealed, and all data were compiled according to the requirements of the Japanese Ministry of Health, Labour and Welfare, which is dedicated to privacy, information technology, and civil rights. Based on the Japanese legislation, the need for informed consent was waived. The study protocol was approved by the Research Ethics Committee of Chiba University School of Medicine (approval number: 2,584). Since 2009, all survivors have provided written informed consent for a prospective cohort study (approval number 826). For patients who died before 2008, written informed consent was not required, in line with the guidelines for retrospective studies in Japan and in accordance with the criteria of the ethics committee of Chiba University Hospital. The study database was anonymized and all experiments were performed in accordance with the relevant guidelines and regulations.

### Patients

**Fig. 1:**
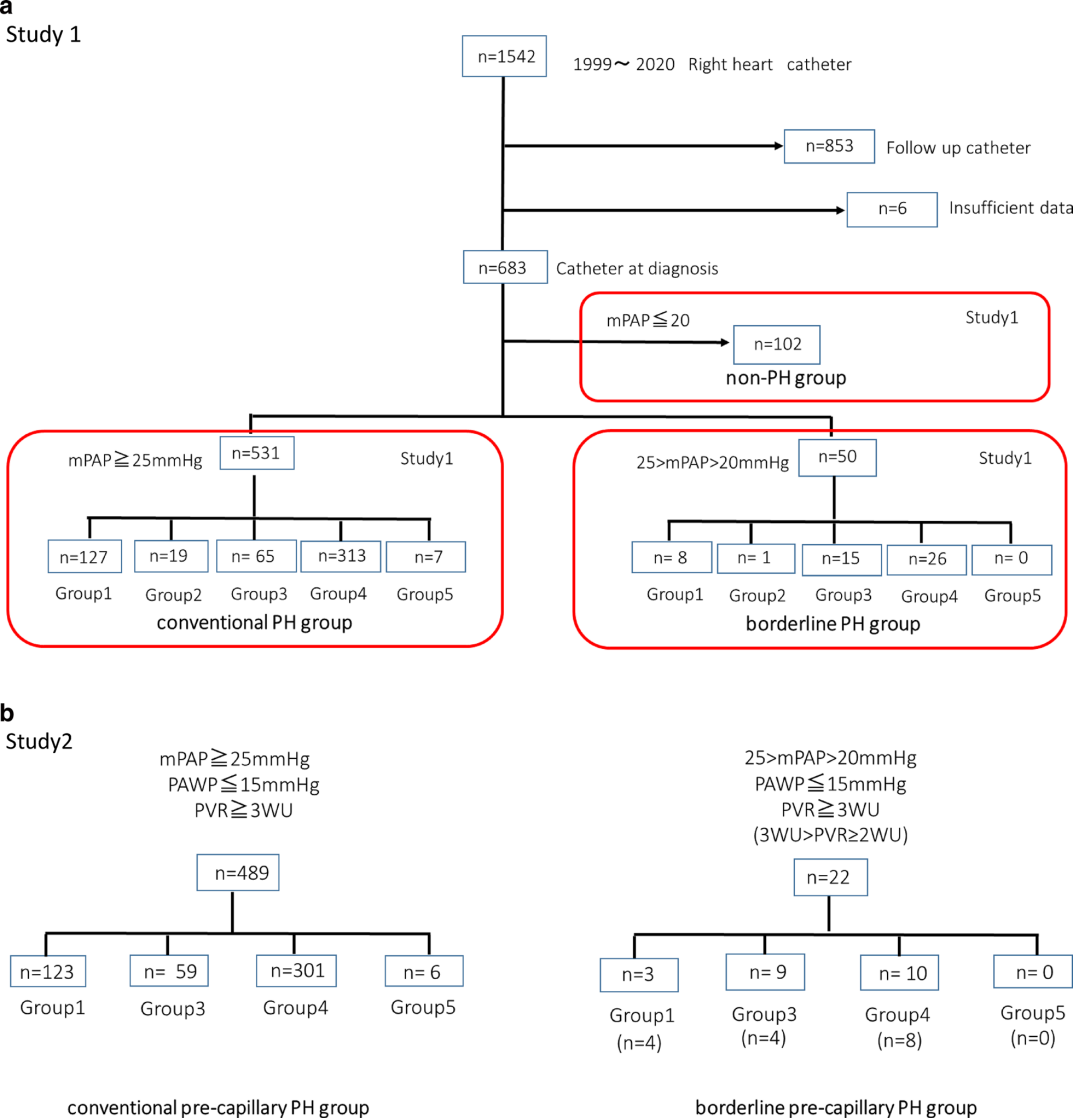
Selection of patients. **a** Among 1542 cases of right heart catheterization (RHC) handled at the Chiba University, Japan, we enrolled 683 patients who underwent the first RHC. Among these, 531 patients had mPAP ≥ 25 mmHg (conventional PH group), and 50 patients had 25 > mPAP > 20 mmHg (borderline PH group). There were 102 patients with mPAP < 20 mmHg (non-PH group). PH, pulmonary hypertension; mPAP, mean pulmonary arterial pressure. **b** We chose patients with PH and pulmonary vascular disease, namely those with pre-capillary PH (mPAP > 20 mmHg with PAWP ≤ 15 mmHg and PVR ≥ 3 WU). Among the patients with pre-capillary PH (n = 501), 489 had mPAP ≥ 25 mmHg (conventional pre-capillary group) and 22 patients had 25 > mPAP > 20 mmHg (borderline pre-capillary group). PH, pulmonary hypertension; mPAP, mean pulmonary arterial pressure; PAWP, pulmonary arterial wedge pressure; PVR, pulmonary vascular resistance; WU, Wood units

Our patients were mainly referred from other hospitals or other departments of Chiba University Hospital. Our PH center is associated with respiratory medicine. Therefore, patients with respiratory diseases suspected of PH during follow-up were also included. From among them, we conducted a retrospective analysis of the data from patients who had undergone RHC. Indication criteria for RHC were as follows: RHC was conducted when a patient’s hypoxia or exercise limitation was considered to have stemmed from PH, and the outcome of RHC seemed to have influenced the patient’s treatment, including their eligibility for clinical trials. Among patients with respiratory diseases, RHC was conducted if transplantation was indicated or when RHC would be beneficial. Among the 1542 patients who underwent RHC between 1999 and 2020 at Chiba University, we enrolled 683 patients who were catheterized for the first time at the first diagnosis. The reason for choosing patients from 1999 was because this was the year epoprostenol was approved in Japan; since then, patients have been prescribed selective pulmonary vasodilators. Among these, 531 patients had mPAP ≥ 25 mmHg (conventional PH group) and 50 patients had mPAP ranging from 20 to 24 mmHg (borderline PH group). The non-PH group consisted of 102 patients with mPAP < 20 mmHg (Fig. 1a). We then chose patients with PH accompanied with pulmonary vascular disease who met the criteria for pre-capillary PH (mPAP > 20 mmHg with PAWP ≤ 15 mmHg and PVR ≥ 3WU). Among the patients with pre-capillary PH (n = 511), mPAP ≥ 25 mmHg was observed in 489 (conventional pre-capillary PH group) and 25 > mPAP > 20 mmHg was observed in 22 patients (borderline pre-capillary PH group) (Fig. 1b). We also categorized our cohort into Group 1–5 according to the Nice 2018 classification [1]; the patients were allocated to the groups after evaluation by two pulmonologists. Despite following the new Nice 2018 recommendation, classifying Group 1 and 3 was occasionally difficult, especially when judging morphological or physiological severity.

For Study 1, we compared the characteristics of the conventional PH, borderline PH, and non-PH groups. The non-PH group was used as a reference since the main aim was to clarify the characteristics of patients diagnosed with PH based on the new definition of pre-capillary PH.

For Study 2, to focus on the pre-capillary nature of PH, the characteristics and survival of the conventional pre-capillary PH and borderline pre-capillary PH groups were compared. For Group 3, the PH prognostic factors were also examined.

Additionally, we compared the survival between the patients with borderline pre-capillary PH (25 > mPAP > 20 mmHg and PAWP ≤ 15 mmHg) with PVR ≥ 3WU and those with 2WU ≤ PVR < 3WU.

With respect to survival, all-cause mortality or lung transplantation was determined in all participants. We excluded patients with a history of PEA as it has been shown to improve the prognosis of patients with chronic thromboembolic pulmonary hypertension [4].

The follow-up data of RHC in the borderline PH and borderline pre-capillary PH groups was also examined.

### Statistical analysis

We used univariate regression analysis to evaluate the baseline characteristics in each group. Student’s t-tests and chi-square tests were used to compare continuous variables and categorical variables, respectively. The differences among the three groups were evaluated using one-way analysis of variance (ANOVA). The results are displayed as mean ± standard deviation or median (interquartile range) for continuous variables, and the number (%) for categorical variables. The Kaplan–Meier method, log‐rank test, and Cox proportional hazards model were used to analyze the mortality and prognostic factors. Multivariate logistic regression analysis was used to identify the factors contributing to poor outcomes. A p-value < 0.05 was considered statistically significant. All analyses were performed using the JMP Pro software 13.2.0, Japanese version (SAS Institute Inc).

## Results

### Conventional PH and borderline PH group (Study 1)

Among the patients who underwent RHC for the first time (n = 683), the number of patients in the conventional PH group was 531 (77.7%), and that in the borderline PH group was 50 (8.6% of total PH patients) (Fig. 1a).

Most of the patients with borderline PH were in Group 3 (30%) and Group 4 (52%) (Table 1). However, according to the clinical classification, most of the patients with borderline PH among the total patients in their specific Groups were in Group 3 (15%), and only 6.8% were in Group 4 (Table 1).

**Table 1:** Demographic data (Study 1: Conventional PH and borderline PH group, including post-capillary PH)

Group	Conventional PH			Borderline PH			Non-PH			Total		
	n	(%)^#^	*% *^*#*^	n	(%) ^#^	*% *^*#*^	n	(%) ^#^	*% *^*#*^	n	(%) ^#^	*% *^*#*^
1	127	*23.9*	81.4	8	*16.0*	5.1	21	*20.6*	13.5	156	*22.8*	100
2	19	*3.6*	90.5	1	*2.0*	4.8	1	*1.0*	4.8	21	*3.1*	100
3	65	*12.2*	65.0	15	*30.0*	15.0	20	*19.6*	20.0	100	*14.6*	100
4	313	*58.9*	82.4	26	*52.0*	6.8	41	*40.2*	10.8	380	*55.6*	100
5	7	*1.3*	87.5	0	*0.0*	0.0	1	*1.0*	12.5	8	*1.2*	100
Unclassified	0	*0.0*	0.0	0	*0.0*	0.0	18	*17.6*	100.0	18	*2.6*	100
All	531	*100.0*	77.7	50	*100.0*	7.3	102	*100.0*	14.9	683	*100.0*	100

*PH* pulmonary hypertension

^#^(%) each clinical classification group in all groups; * % of conventional PH group, borderline PH group, non-PH group among each clinical classification group

Detailed analysis, based on etiology, showed that hemodynamics, alveolar-arterial oxygen difference (AaDO_2_), gas exchange impairment, and 6-min walk distance were worst in the conventional group (Table 2). In addition, the partial pressure of arterial carbon dioxide (PaCO_2_) was the lowest in the conventional PH group. In Group 3, no significant differences in parameters of the ventilatory function were observed among the conventional, borderline PH, and non-PH groups.

**Table 2:** Baseline characteristics (Study 1; Conventional PH and borderline PH group, including post-capillary PH)

Total		Conventional				Borderline				Non-PH			p-value
	n				n				n				
Age	531	56.7	±	15.2	50	56.2	±	15.8	102	57.8	±	16.1	0.7591
Sex(F/M)	531	373	/	158	50	29	/	21	102	70	/	32	0.2152
mPAP(mmHg)	531	42.9	±	11.8	50	22.6	±	1.2	102	16.2	±	3.2	< 0.0001
PVR (WU)	531	8.7	±	4.7	50	3.0	±	1.3	102	2.2	±	0.9	< 0.0001
PAWP(mmHg)	531	8.1	±	3.8	50	8.0	±	3.9	102	5.9	±	3.0	< 0.0001
CO(L/min)	531	4.5	±	1.5	50	5.4	±	1.8	102	5.1	±	1.4	< 0.0001
6MWD(m)	413	362.0	±	105.3	32	410.4	±	105.5	51	429.0	±	112.6	< 0.0001
%VC	482	85.4	±	21.5	39	88.6	±	26.0	86	88.9	±	24.3	0.3056
FEV1.0%	182	75.5	±	11.4	39	77.6	±	18.5	86	78.0	±	15.0	0.176
%DLCO/VA	455	76.5	±	27.4	38	74.1	±	25.2	79	85.1	±	31.1	0.0297
PaO_2_(mmHg)	517	65.3	±	22.1	50	74.3	±	13.0	99	82.5	±	17.6	< 0.0001
PaCO_2_(mmHg)	517	38.5	±	6.4	50	40.6	±	7.2	99	40.2	±	5.3	0.0051
P_V_O_2_(mmHg)	515	34.7	±	4.9	50	38.1	±	4.8	99	40.2	±	7.9	< 0.0001
O_2_ administration( +)		75(14.1%)				3(6.0%)				8(7.8%)			0.0536
AaDo_2_(mmHg)	516	39.0	±	25.0	50	17.6	±	11.1	99	19.6	±	16.5	< 0.0001
WHO-FC (I/II/III/IV)		(6/259/255/11)				(0/37/13/0)				(16/62/23/1)			< 0.0001
Vasodilators( ±)	531	316	/	215	50	8	/	42	102	2	/	100	< 0.0001
*Group 1*													
Age	127	48.6	±	18.1	8	49.5	±	6.2	21	58.7	±	3.9	0.0546
Sex(F/M)	127	103	/	24	8	5	/	3	21	20	/	1	0.0346
mPAP(mmHg)	127	44.3	±	12.1	8	22.8	±	1.3	21	16.6	±	2.8	< 0.0001
PVR (WU)	127	8.7	±	4.8	8	3.0	±	1.3	21	2.2	±	1.1	< 0.0001
PAWP(mmHg)	127	7.5	±	4.5	8	7.5	±	4.5	21	5.8	±	3.1	0.0337
CO(L/min)	127	4.8	±	1.7	8	6.1	±	3.1	21	5.3	±	1.5	0.1076
6MWD(m)	97	398.5	±	108.6	6	434.2	±	61.6	14	455.2	±	111.2	0.152
VC,% predicted	115	85.2	±	16.9	6	89.8	±	9.8	19	86.1	±	14.9	0.7914
FEV1.0,% predicted	115	78.9	±	9.8	6	79.2	±	8.9	19	82.1	±	10.1	0.432
DLCO/VA,% predicted	111	74.7	±	25.7	6	76.3	±	16.5	18	81.2	±	39.3	0.659
PaO_2_(mmHg)	125	72.6	±	18.9	8	75.9	±	13.8	21	87.7	±	15.8	0.0018
PaCO_2_(mmHg)	125	36.6	±	5.3	8	39.6	±	1.2	21	40.7	±	6.6	0.0037
P_V_O_2_(mmHg)	123	37.7	±	5.1	8	41.8	±	8.2	21	42.3	±	5.5	0.0005
O_2_ administration( +)		18(14.2%)				0(0.0%)				0(0.0%)			0.019
AaDo_2_(mmHg)	125	33.8	±	19.3	8	27.1	±	13.8	21	14.0	±	12.4	< 0.0001
WHO-FC (I/II/III/IV)		(3/83/40/1)				(0/7/1/0)				(1/18/2/0)			0.2859
Vasodilators( ±)	127	95	94	33	8	4	/	4	21	1	/	20	< 0.0001
Underlying diseases			(%)				(%)				(%)		
IPAH/HPAH/PVOD/PCH		57	44.9			-	-			-	-		
CTD		39	30.7			4	50.0			8	38.1		
Congenital		19	15.0			2	25.0			3	14.3		
Portal hypertension		12	9.4			1	12.5			1	4.8		
drug/HIV		2	1.6			0	0.0			0	0.0		
unknown		–	–			1	12.5			9	42.9		
*Group 3*													
Age	65	61.5	±	13.4	15	59.7	±	18.7	20	60.1	±	13.8	0.8723
Sex(F/M)	65	33	/	32	15	6	/	9	20	5	/	15	0.0321
mPAP(mmHg)	65	35.7	±	10.8	15	22.4	±	1.4	20	16.0	±	3.4	< 0.0001
PVR (WU)	65	7.0	±	5.2	15	3.3	±	1.2	20	2.5	±	0.9	< 0.0001
PAWP(mmHg)	65	7.1	±	3.7	15	7.1	±	3.7	20	4.8	±	2.6	0.0016
CO(L/min)	65	4.7	±	1.6	15	5.2	±	1.8	20	47.0	±	1.2	0.5076
6MWD(m)	38	281.5	±	87.2	10	310.2	±	121.7	14	376.1	±	99.4	0.01
VC,% predicted	58	58.7	±	24.0	13	68.3	±	28.6	20	71.5	±	26.6	0.1134
FEV1.0,% predicted	58	73.2	±	18.9	13	75.4	±	30.9	20	69.9	±	24.2	0.7678
DLCO/VA,% predicted	47	43.3	±	29.1	12	53.3	±	27.4	17	69.3	±	22.4	0.0052
PaO_2_(mmHg)	59	66.1	±	38.0	15	67.0	±	14.6	19	81.3	±	18.4	0.1991
PaCO_2_(mmHg)	59	46.8	±	10.5	15	45.6	±	9.9	19	42.2	±	5.8	0.1983
P_V_O_2_(mmHg)	59	35.3	±	4.4	15	35.5	±	2.9	19	38.8	±	5.4	0.0134
O_2_ administration( +)	65	1(6.7%)				18(27.7%)				3(15.0%)			0.1096
AaDo_2_(mmHg)	58	28.9	±	47.3	15	29.0	±	13.3	19	18.7	±	17.7	0.5993
WHO-FC (I/II/III/IV)		(0/14/49/2)				(0/6/9/0)				(1/4/14/1)			0.3784
Vasodilators	65	35	/	30	15	0	/	15	20	0	/	20	< 0.0001
Underlying diseases			(%)				(%)				(%)		
IP		38	58.5			7	46.7			8	40.0		
COPD		13	20.0			5	33.3			7	35.0		
BE		8	12.3			1	6.7			0	0.0		
Others		6	9.2			2	13.3			1	5.0		
*Group 4*													
Age	313	58.3	±	13.1	26	55.5	±	14.4	41	57.0	±	17.4	0.5267
Sex(F/M)	313	223	/	90	26	18	/	8	41	29	/	12	0.9757
mPAP (mmHg)	313	44.3	±	11.4	26	22.7	±	1.2	41	16.5	±	3.0	< 0.0001
PVR (WU)	313	9.4	±	4.5	26	3.0	±	1.3	41	2.2	±	0.8	< 0.0001
PAWP (mmHg)	313	7.6	±	3.2	26	8.3	±	3.5	41	5.9	±	2.7	0.0018
CO (L/min)	313	4.3	±	1.1	26	5.2	±	1.3	41	5.0	±	1.1	< 0.0001
6MWD (m)	264	359.4	±	100.2	15	465.7	±	54.8	15	451.5	±	119.5	< 0.0002
VC,% predicted	290	91.8	±	18.0	19	103.4	±	17.1	31	101.3	±	21.1	0.0012
FEV1.0,% predicted	290	75.0	±	9.4	19	79.0	±	7.8	31	78.2	±	9.1	0.0631
DLCO/VA,% predicted	278	82.5	±	23.8	19	85.2	±	18.0	30	96.9	±	19.2	0.0054
PaO_2_ (mmHg)	308	60.9	±	18.0	26	78.2	±	10.3	40	78.5	±	15.8	< 0.0001
PaCO_2_ (mmHg)	308	37.6	±	4.4	26	38.5	±	4.4	40	39.7	±	4.7	0.0114
P_V_O_2_ (mmHg)	308	33.3	±	4.3	26	38.7	±	3.4	40	39.2	±	3.4	< 0.0001
O_2_ administration(+)		36(9.5%)				2(7.7%)				0(0.0%)			0.0115
AaDo_2_(mmHg)	308	44.4	±	18.9	26	26.1	±	8.4	40	24.3	±	14.8	< 0.0001
WHO-FC (I/II/III/IV)		(3/147/156/7)				(0/24/2/0)				(8/30/3/0)			< 0.0001
Vasodilators	313	177	/	136	26	4	/	22	41	1	/	40	< 0.0001
PEA	313	158	/	155	26	2	/	24	41	1	/	40	< 0.0001
BPA	313	53	/	260	26	0	/	26	41	0	/	41	< 0.0001
Underlying diseases			(%)				(%)				(%)		
PE		289	92.3			22	84.6			35	85.4		
Pulmonary stenosis		22	7.0			4	15.4			5	12.2		
Others		2	0.6			0	0.0			1	2.4		

mPAP, mean pulmonary arterial pressure; PVR, pulmonary vascular resistance; PAWP, pulmonary arterial wedge pressure; CO, cardiac output; 6MWD, 6-min walk distance; %VC, percent vital capacity; FEV1.0%, percent predicted forced expiratory volume in one second; %DLCO/VA, diffusing capacity of carbon monoxide by the alveolar volume; PaO_2_, partial pressure of arterial oxygen; PaCO_2_, partial pressure of arterial carbon dioxide; PvO_2_, mixed venous oxygen tension; AaDO_2_, alveolar-arterial oxygen difference; WHO-FC, World Health Organization Functional Class; IPAH, idiopathic pulmonary hypertension; HPAH, hereditary pulmonary hypertension; PVOD, pulmonary veno-occlusive disease; PCH, pulmonary capillary hemangiomatosis; CTD, connective tissue disease; ILD, interstitial lung disease; COPD, chronic obstructive pulmonary disease; BE, bronchiectasis; PEA, pulmonary endarterectomy; PE, pulmonary embolism

**Fig. 2:**
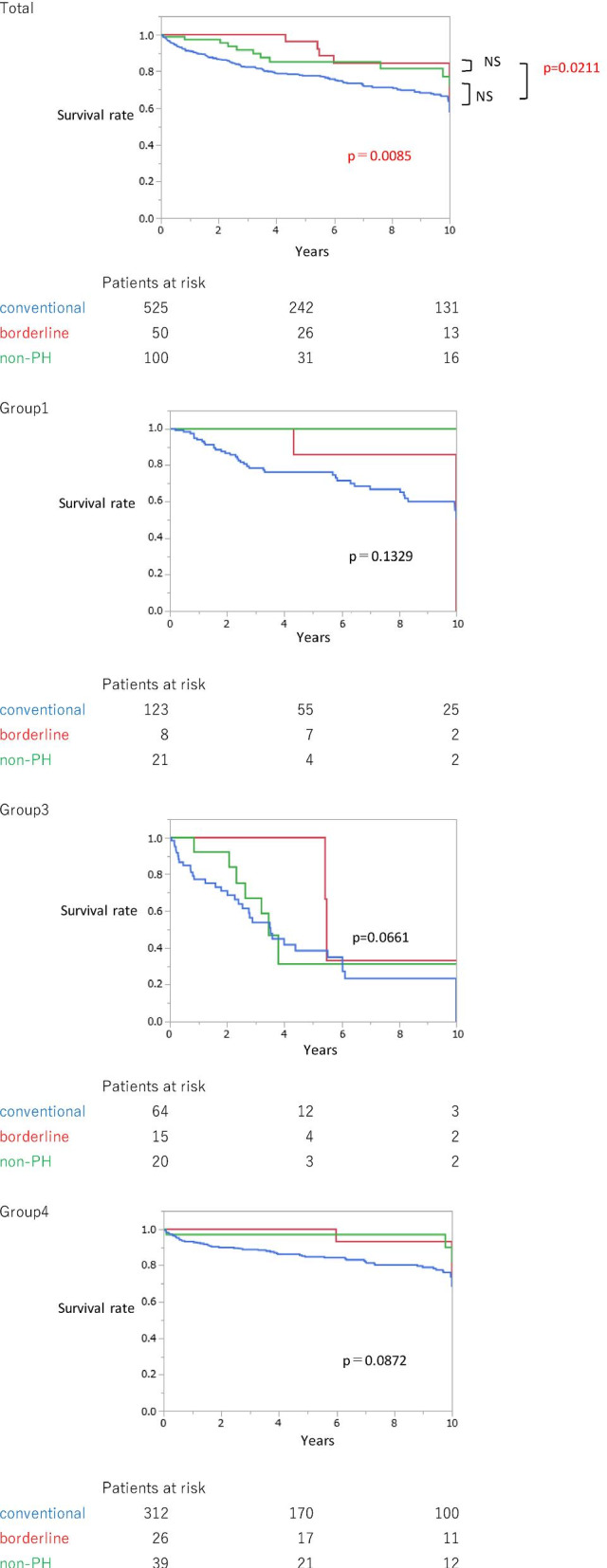
Survival (Study 1: Conventional PH and borderline PH group including post-capillary PH). The survival of the conventional PH group was the worst among the 3 groups (p = 0.0085). There was no significant difference in the survival between the borderline group and the non-PH group. PH, pulmonary hypertension

Next, we compared the survival of the conventional PH, borderline, and non-PH groups (Fig. 2). The survival of the conventional PH group was worse than that of the borderline group, and the worst among all three groups. Analogical tendencies were observed in Groups 1 and 4. However, in Group 3, the 10-year survival of all groups was < 40%. Group 3 showed poor prognosis, even in the non-PH group.

We divided the patients into two sub-groups according to the time of diagnosis (diagnosed in 1999–2009 or 2010–2020), since we recently tended to perform RHC only in patients who may benefit from treatment using vasodilators. The ratio of patients in the non-PH group diagnosed in 1999–2009 was higher than that diagnosed in 2010 (Table 3). Moreover, we analyzed the cause of death among the patients in Group 3. The number of patients who died due to malignant disease or who underwent lung transplantation was higher in the non-PH group than in the PH group (Table 4).

**Table 3:** Time of diagnosis in Group 3

	~ 2009		~ 2010	
	n	%	n	%
Conventional	9	40.9	56	71.8
Borderline	4	18.2	11	14.1
Non-PH	9	40.9	11	14.1
Total	22		78	

p = 0.0168

**Table 4:** Causes of death or lung transplantation in Group 3

	RHF		Lung disease		Malignancy		Lung transplantation		Others		Total
	n	%	n	%	n	%	n	%	n	%	
Conventional	10	29.4	14	41.2	1	2.9	2	5.9	7	20.6	34
Borderline	0	0.0	1	50.0	0	0.0	0	0.0	1	50.0	2
Non-PH	0	0.0	3	42.9	1	14.3	2	28.6	1	14.3	7

PH, pulmonary hypertension; RHF, right heart failure

### Characteristics and survival of pre-capillary PH patients (Study 2)

The number of patients in the conventional pre-capillary PH group was 489 (71.6%). Twenty-two patients (3.2% of the total patients, including the patients with non-PH; 4.3% of the patients with total pre-capillary PH) were included in the borderline pre-capillary PH group (Fig. 1b and Table 5). Similar to Study 1, most of the patients with borderline pre-capillary PH belonged to Groups 3 (40.9%) and 4 (45.5%). However, among the total patients with pre-capillary PH, most of the patients with borderline pre-capillary PH belonged to Group 3 (13.2%), and only 3.2% patients belonged to Group 4 (Table 5).

**Table 5:** Demographic data (Study 2: Conventional PH and borderline PH group in pre-capillary PH)

	Conventional pre-capillary PH			Borderline pre-capillary PH			Total pre-capillary PH		
Group	n	(%) ^#^	*% *^*#*^	n	(%) ^#^	*% *^*#*^	n	(%) ^#^	*% *^*#*^
1	123	*25.2*	97.6	3	2.4	*13.6*	126	*24.7*	100
3	59	*12.1*	86.8	9	13.2	*40.9*	68	*13.3*	100
4	301	*61.6*	96.8	10	3.2	*45.5*	311	*60.9*	100
5	6	*1.2*	100.0	0	0.0	*0.0*	6	*1.2*	100
Total	489	*100.0*	95.7	22	4.3	*100.0*	511	*100.0*	100

PH, pulmonary hypertension

^#^ (%): each clinical classification group in all groups; *%: conventional pre-capillary PH group and borderline pre-capillary PH group among each clinical classification group

Regarding baseline characteristics, in addition to hemodynamics, partial pressure of arterial oxygen (PaO_2_), partial pressure of mixed venous oxygen (PvO_2_), and AaDO_2_ in the conventional pre-capillary PH group were significantly worse than those in the borderline pre-capillary PH group. In addition, PaCO_2_ was significantly lower in the conventional pre-capillary PH group. There was no significant difference in the ventilatory function between the two groups.

Focusing on each etiological group, the conventional pre-capillary PH group showed more severe hemodynamics and gas exchange impairment than the borderline pre-capillary PH group in Groups 1, 3, and 4. A significantly lower PaCO_2_ in the conventional pre-capillary PH group was observed only in Group 1 relative to that in the other Groups. Even in Group 3, there was no significant difference in the ventilatory function parameters between the two groups (Table 6).

**Table 6:** Baseline characteristics (Study 2: Conventional PH and borderline PH group in pre-capillary PH)

Total	N	Conventional pre-capillry PH			n	Borderline pre-capillary PH			p-value
Age	489	56.1	±	15.2	22	58.2	±	13.9	0.5342
Sex(F/M)	489	347	/	142	22	15	/	7	0.7808
mPAP(mmHg)	489	43.7	±	11.7	22	23.0	±	1.2	< 0.0001
PVR (WU)	489	9.2	±	4.6	22	4.2	±	0.9	< 0.0001
PAWP(mmHg)	489	7.5	±	3.1	22	5.7	±	2.9	0.0057
CO(L/min)	489	4.3	±	1.2	22	4.2	±	0.9	0.5689
6MWD(m)	449	360.1	±	104.7	12	403.2	±	11.3	0.1704
VC,% predicted	444	86.7	±	21.2	17	86.0	±	28.6	0.8827
FEV1.0,% predicted	444	75.8	±	10.8	17	73.9	±	17.3	0.4922
DLCO/VA,% predicted	420	75.8	±	27.3	16	70.7	±	21.8	0.4577
PaO_2_(mmHg)	476	64.4	±	21.5	22	74.6	±	14.2	0.0281
PaCO_2_(mmHg)	476	38.2	±	6.1	22	41.4	±	9.3	0.0212
P_V_O_2_(mmHg)	475	34.4	±	4.7	22	37.1	±	3.7	0.0087
O_2_ administration( +)			71(14.5%)				1(4.6%)		0.1348
AaDo_2_(mmHg)	475	40.3	±	23.8	22	26.3	±	10.3	0.0063
WHO-FC (I/II/III/IV)		(6/231/241/11)				(0/17/5/0)			0.0344
Vasodilators( ±)	489	301	/	188	22	5	/	17	< 0.0001
*Group 1*									
Age	123	47.9	±	17.8	3	51.0	±	66.0	0.0357
Sex(F/M)	123	99	/	24	3	2	/	1	0.1417
mPAP(mmHg)	123	44.8	±	12.0	3	23.3	±	0.6	< 0.0001
PVR (WU)	123	9.0	±	4.7	3	4.4	±	0.5	< 0.0001
PAWP(mmHg)	123	7.7	±	3.1	3	7.0	±	1.7	0.0359
CO(L/min)	123	4.7	±	1.5	3	3.8	±	0.7	0.1295
6MWD(m)	95	398.0	±	107.4	3	472.0	±	61.5	0.2394
VC,% predicted	111	85.6	±	16.9	3	91.4	±	9.5	0.8324
FEV1.0,% predicted	111	79.1	±	9.4	3	76.9	±	9.9	0.4183
DLCO/VA,% predicted	107	73.4	±	23.9	3	74.4	±	12.8	0.5192
PaO_2_(mmHg)	121	72.6	±	17.9	3	82.3	±	3.6	0.0013
PaCO_2_(mmHg)	121	36.6	±	5.3	3	39.3	±	0.7	0.0072
P_V_O_2_(mmHg)	120	37.3	±	4.8	3	39.8	±	1.4	0.0003
O_2_ administration( +)			17(13.8%)				0(0%)		0.3479
AaDo_2_(mmHg)	121	33.9	±	19.2	3	21.0	±	4.4	< 0.0001
WHO-FC (I/II/III/IV)	123		(3/79/40/1)		3		(0/3/0/0)		0.4546
Vasodilators( ±)	123	92	/	31	3	2	/	1	< 0.0001
Underlying diseases			(%)				(%)		
IPAH/HPAH/PVOD/PCH		55	44.7			-	-		
CTD		38	30.9			1	33.3		
Congenital		16	13.0			1	33.3		
Portal hypertension		12	9.8			0	0.0		
drug/HIV		2	1.6			0	0.0		
unknown			–			1	33.3		
*Group 3*									
Age	59	62.3	±	13.2	9	63.3	±	13.5	0.8404
Sex(F/M)	59	28	/	31	9	5	/	4	0.6506
mPAP(mmHg)	59	36.5	±	11.0	9	22.4	±	1.3	0.0003
PVR (WU)	59	7.4	±	5.2	9	4.0	±	0.8	0.0546
PAWP(mmHg)	59	4.5	±	3.2	9	5.7	±	3.7	0.12
CO(L/min)	59	4.4	±	1.2	9	4.3	±	1.2	0.8225
6MWD(m)	36	281.2	±	89.5	5	317.6	±	107.4	0.4098
VC,% predicted	52	59.8	±	24.2	7	66.9	±	33.5	0.4882
FEV1.0,% predicted	52	73.6	±	17.2	7	68.6	±	24.5	0.498
DLCO/VA,% predicted	43	42.0	±	30.0	6	51.9	±	18.7	0.4353
PaO_2_(mmHg)	53	64.4	±	35.4	9	65.8	±	15.6	0.9052
PaCO_2_(mmHg)	53	46.2	±	9.6	9	47.0	±	12.1	0.8248
P_V_O_2_(mmHg)	53	34.9	±	4.0	9	35.1	±	3.0	0.9152
O_2_ administration( +)			17(28.8%)				1(11.1%)		0.2265
AaDo_2_(mmHg)	52	31.4	±	42.9	9	28.5	±	13.7	0.8458
WHO-FC (I/II/III/IV)			(0/11/46/2)				(0/5/4/0)		0.0671
Vasodilators( ±)	59	35	/	24	9	0	/	9	0.0001
Underlying diseases			(%)				(%)		
ILD		36	61.0			4	44.4		
COPD		13	22.0			3	33.3		
BE		6	10.2			1	11.1		
Others		4	6.8			1	11.1		
*Group 4*									
Age	301	58.2	±	13.1	10	55.7	±	15.2	0.5475
Sex(F/M)	301	216	/	85	10	8	/	2	0.5551
mPAP(mmHg)	301	44.7	±	11.2	10	23.4	±	0.7	< 0.0001
PVR (WU)	301	9.6	±	4.4	10	4.3	±	1.1	0.0002
PAWP(mmHg)	301	7.5	±	3.1	10	5.3	±	2.4	0.0269
CO(L/min)	301	4.2	±	1	10	4.2	±	0.6	0.8073
6MWD(m)	254	357.8	±	99.4	4	458.5	±	80.4	0.0449
VC,% predicted	278	92.2	±	18.1	7	102.7	±	16	0.1311
FEV1.0,% predicted	278	75	±	9.3	7	78	±	10.2	0.4101
DLCO/VA,% predicted	267	82.3	±	24	7	85.2	±	15.6	0.7473
PaO_2_(mmHg)	296	60.7	±	18.2	10	80.1	±	11.1	0.0009
PaCO_2_(mmHg)	296	37.5	±	4.4	10	37	±	4.2	0.7296
P_V_O_2_(mmHg)	296	33.1	±	1.2	10	38.2	±	3.9	0.0002
O_2_ administration( +)			34 (11.3%)				0(0%)		0.1248
AaDo_2_(mmHg)	296	44.8	±	19.1	10	25.9	±	8.1	0.002
WHO-FC (I/II/III/IV)		(3/138/153/7)					(0/9/1/0)		0.0345
Vasodilators( ±)	301	168	/	133	10	3	/	7	0.1049
PEA	301	156	/	145	10	0	/	14	0.0002
BPA	301	52	/	249	10	0	/	14	0.0536
Underlying diseases			(%)				(%)		
PE		279	92.7			9	90.0		
Pulmonary stenosis		20	6.6			1	10.0		
Others		2	0.7			0	0.0		

Data are expressed as mean ± SD or n (%). mPAP, mean pulmonary arterial pressure; PVR, pulmonary vascular resistance; PAWP, pulmonary arterial wedge pressure; CO, cardiac output; 6MWD, 6-min walk distance; %VC, percent vital capacity; FEV1.0%, percent predicted forced expiratory volume in one second; %DLCO/VA, diffusing capacity of carbon monoxide by the alveolar volume; PaO_2_, partial pressure of arterial oxygen; PaCO_2_, partial pressure of arterial oxygen; PvO_2_, mixed venous oxygen tension; AaDO_2_, alveolar-arterial oxygen difference; WHO-FC, World Health Organization Functional Class; IPAH, idiopathic pulmonary hypertension; HPAH, hereditary pulmonary hypertension; PVOD, pulmonary veno-occlusive disease; PCH, pulmonary capillary hemangiomatosis; CTD, connective tissue disease; ILD, interstitial lung disease; COPD, chronic obstructive pulmonary disease; BE, bronchiectasis; PEA, pulmonary endarterectomy; PE, pulmonary embolism

**Fig. 3:**
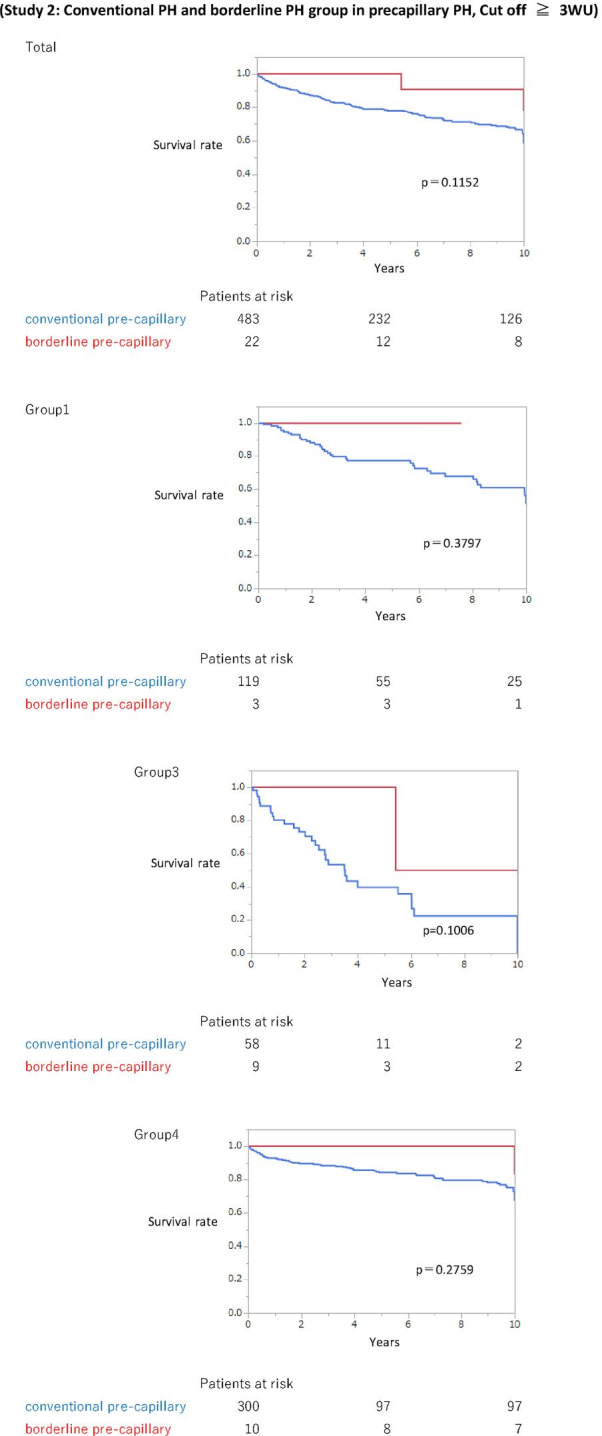
Survival (Study 2: Conventional PH and borderline PH group in pre-capillary PH, cut-off ≥ 3 WU). The survival of the conventional pre-capillary PH group was worse than that of the borderline pre-capillary PH group; however, the difference was not statistically significant. The same tendency was observed in Groups 1, 3 and 4. In Group 3, the 5-year and 10-year survival rates in the conventional pre-capillary PH population were 39.8% and 22.4%, respectively. The survival rates in the borderline pre-capillary PH group were 100% and 50%, respectively. PH, pulmonary hypertension; WU, Wood units

Furthermore, the survival of the conventional pre-capillary PH group was worse than that of the borderline pre-capillary PH group; however, it did not reach statistical significance. The same tendency was observed in Group 1, 3, and 4 (Fig. 3). The 10-year-survival was the worst in Group 3 in the conventional PH group (Group 1, 50.1%; Group 3, 0.00%; Group 4, 68.0%) and the borderline pre-capillary PH group (Group 1, 50.9%; Group 3, 0.0%; Group 4, 67.0%). Age and conventional PH vs. borderline PH were poor prognostic factors; however, no parameter was statistically significant (Table 7).

**Table 7:** Factors affecting the prognosis of Group 3 pre-capillary PH

Factors	Univariate crude hazard ratio (95% CI)	p-value	Multivariate hazard ratio (95% CI)	p-value
Age	0.973 (0.949–1.000)	0.052	0.992 (0.947–1.000)	0.051
Hemodynaics classification (Conventional pre-capillary/Borderline pre-capillary)	4.690 (0.623–35.284)	0.055	4.265 (0.547–33.238)	0.09
Time of diagnosis (~ 2010/2010 ~)	0.846 (0.317–2.259)	0.073	1.216 (0.416–3.551)	0.716

PVR, pulmonary vascular resistance

**Fig. 4:**
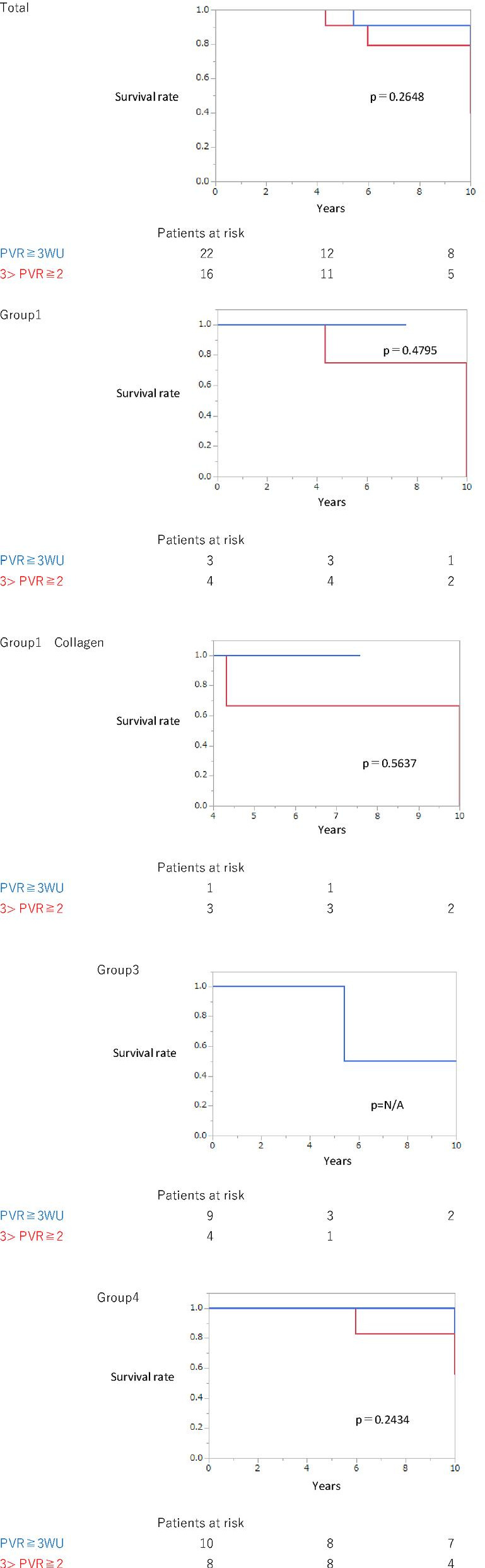
Survival (Pre-capillary PH, PVR Cut-off: PVR ≥ 3 WU vs. 3 > PVR ≥ 2 WU). There was no significant difference between the patients with pre-capillary PH with PVR ≥ 3 WU and those with 3 > PVR ≥ 2 WU. PH, pulmonary hypertension; WU, Wood units; PVR, pulmonary vascular resistance

There was no significant difference in survival between the patients with borderline pre-capillary PH (25 > mPAP > 20 mmHg and PAWP ≤ 15 mmHg) with PVR ≥ 3 WU (n = 22) and those with 2 ≤ PVR < 3 WU (n = 16) (Fig. 4). Among them, five patients died; however, none of them succumbed to right heart failure (PVR ≥ 3: 1, pneumonia; 2 ≤ PVR < 3WU: 4, malignancy).

### Follow-up RHC data of patients with borderline PH and those with borderline pre-capillary PH

Among the patients with borderline PH, follow-up RHC was performed when the clinical condition was considered to worsen. Among 50 patients with borderline PH and 22 with borderline pre-capillary PH, only five patients underwent follow-up RHC (borderline PH = 4; borderline pre-capillary PH = 1), and only two patients were prescribed pulmonary vasodilators (borderline PH = 1; borderline pre-capillary PH = 1). Only one patient’s condition improved after treatment (Table 8).

**Table 8:** Follow-up data of borderline PH, according to definition of PVR cut-off ≥ 3 WU

Group	Group of PH	Background disease	Age at diagnosis	RHC at diagnosis			Desition after first RHC	RHC at final follow up			Desition after final RHC	Outcome
				mPAP (mmHg)	PVR (WU)	PCWP (mmHg)		mPAP (mmHg)	PVR (WU)	PCWP (mmHg)		
Boderline PH	1	SSc	37	22	2.6	5	Follow up	37	2.6	9	Ambrisentan	Deceased because of lung cancer in 14 month
Boderline PH	3	COPD	65	23	2.6	5	Follow up	35	3.7	4	Treatment of COPD	Became feasible because of COPD
Boderline PH	3	SSc + Fibrosis	35	20	1.2	11	Follow up	40	2.9	16	Treatment of left heart failure	Deceased because of left heart failure in 11 month
Boderline PH	4	CTEPH	46	24	2.4	7	Follow up	19	1.1	10	Follow up	Stable
Boderline pre-capillary PH	4	Aortitis	61	24	5.8	4	Follow up	29	7.7	1	Riociguat	Improved after the treatment of riociguat

SSc, Systemic sclerosis; COPD, Chronic obstructive pulmonary disease; CTEPH; Chronic thromboembolic pulmonary hypertension

## Discussion

This is the first study conducted in a PH center in an Asian country to reveal the characteristics and survival of patients diagnosed with pre-capillary PH, according to the new diagnostic criteria proposed at the WSPH 2018. The borderline pre-capillary PH group accounted for 3.2% (22 in 683) of the total patient population who underwent their first RHC, and 4.3% of all patients with pre-capillary PH (22 in 511). The borderline PH group comprised 8.6% (50 in 581) of all patients with PH. Most of the patients in the borderline pre-capillary PH group belonged to Group 3 and 4. The survival of the borderline pre-capillary PH group tended to be better than that of the conventional pre-capillary PH group. Further, the prognosis of PH in Group 3 was the worst among the patients with borderline precapillary PH.

Most of the previous studies have focused on patients with borderline PH (25 > mPAP > 20 mmHg), and not on the pre-capillary nature of PH. In previous studies, the percentage of borderline PH has ranged from 4.2 to 18% among all patients and 4.5–22.6% of all patients with mPAP > 20 mmHg [5–7]. Further, Group 2 PH corresponds to the most common form of conventional PH due to left heart failure [8]. Previous studies have shown a higher percentage of borderline PH when the sample included more patients with cardiac diseases.

Assad et al. showed that among all patients, the percentage of those with mPAP between 19 and 24 mmHg, including non-PH patients (20.1%) was 18%, and that the majority of them belonged to Group 2 due to the presence of cardiovascular disease in > 70% of the patients [5]. Douschan et al. observed that 11.7% of all patients, including those without PH (35.2%), had borderline PH [6]. In their study, 20.3% of patients belonged to Group 2 with overt PH, and patients with borderline PH and those with overt PH showed a higher risk of cardiac disease. However, in these studies, detailed demographic data, including number of patients without PH, were not known [5, 6]. Another study showed that the total percentage of patients with borderline PH was only 4.2%, including non-PH patients (5.7%) [7]. In that study, a relatively lower percentage of left heart disease (16.2%) and a relatively higher percentage of respiratory disease (29.7%) was observed among the patients with borderline PH and those without PH. However, the background status of all patients, including those with overt PH, is not known (Table 9) [7]. Only one study showed the proportion of patients with borderline pre-capillary PH; however, the number was quite small and did not show the prognosis [9].

**Table 9:** Comparison with previous reports regarding the percentage of patients with “borderline pre-capillary PH” or “borderline PH”

	Country	n	% of borderline pre-capillary PH		% of borderline PH	of total PH patients	Associated conditions
			of total patients (including Non-PH)	of total pre-capillary PH patients	of total patients (including Non-PH)		Total
Assad et. al.(2017)[5]	U.S.A	4343	–	–	18.0%	22.6%	CTD:0.9% CAD:71.5% COPD + ILD:18.7%
Douschan et.al.(2018)[6]	Austria	547	–	–	11.7%	18.1%	※Conventional group only Group1:25.5% Group2:20.3% Group3:26.6% Group4:18.3% Group5:9.3%
Gustavo et.al. (2013)[7]	the U.S	1491	–	–	4.2%	4.5%	※Borderline + borerline precapillary only None:30.4% CTD:16.2% Heart disease:16.2% Respiratory disease:29.7
Umit et.al. (2019)[9]	Turkey	58	12.1%	14.0%	Unknown	Unknown	IPAH suspected:43.1% Congenital PH suspected:34.5% Systemic sclerosis: 3.4% Left heart disease and valvular disease:6.9%
Our study	Japan	683	3.2%	4.3%	7.3%	8.6%	Group1:22.8% Group2:3.1% Group3:14.6% Group4:55.6% Group5:1.2%

CTD, connective tissue disease; CAD, coronary artery disease; COPD, chronic obstructive disease; ILD, interstitial lung disease; IPAH, idiopathic pulmonary hypertension; PH, pulmonary hypertension

In our study, the number of patients with borderline PH, including those in PH Groups 1−5, was 8.6% lower than that reported in Assad’s and Douschan’s studies (22.6% and 18.1%, respectively) (Table 9), and most of the total patients belonged to Group 3 (14.6%) and 4 (55.6%). The relatively higher ratio of these groups can be explained by our PH center being associated with respiratory medicine and being one of the high-volume PEA centers in Japan. Furthermore, the proportion of patients in Group 2, which is the most common form, was quite low. In our study, the ratio of borderline PH was relatively high in Group 3. Similarly, a study on 15 patients with severe COPD who underwent lung transplantation also showed a mild elevation of mPAP (20−25 mmHg) in most patients [10]; therefore, the majority of patients with severe lung disease tended to have a mild elevation of mPAP. In contrast, our data showed that the ratio of borderline PH in Group 4 was low. The Papworth hospital study, which was also conducted in a PEA center, reported chronic thromboembolic disease with mPAP < 25 mmHg in only 42 of 1019 patients (4.1%) who underwent PEA [11]. The higher percentage of Group 4 patients in our center may explain the lower percentage of the borderline PH group in total. Overall, the number of patients who met the new diagnostic criteria depended on their background status.

Regarding baseline characteristics, in addition to hemodynamics, the PaO_2_, PvO_2_, and AaDO_2_ were better in the borderline pre-capillary PH group than in the conventional pre-capillary PH group. Lower PaCO_2_ in Group 1 of the conventional PH group might be suggestive of hyperventilation compensating for gas exchange impairment.

Several studies have shown little correlation between ventilatory function and severity of PH in patients with lung disease [12–14]. Similarly, in our study, there was no significant difference in ventilatory function between the conventional and the borderline pre-capillary PH groups in Group 3 (Table 6).

Regarding survival, in Study 1, the survival of the conventional PH group was worse than that of the borderline PH group. Similarly, in Study 2, the survival of the conventional pre-capillary PH group was worse than that of the borderline pre-capillary PH group; however, no significant difference was observed between the borderline PH and non-PH groups.

Previous data has suggested that mild elevation of PH is associated with poor prognosis in idiopathic pulmonary fibrosis [15] or chronic obstructive pulmonary disease [16, 17]. Assad et al. also showed poor prognosis in patients with borderline PH, and the majority of patients seemed to be in Group 2 [5]. Douchan et al. revealed poorer prognosis and increased cardiopulmonary comorbidities in patients with mPAP of 17–26 mmHg than in those with mPAP < 17 mmHg [6]. They chose patients having a similar background status in both the PH and non-PH groups. Although a report including patients with relatively heterogeneous background diseases also showed poor prognosis of patients with borderline PH, the difference in the prognosis between patients with overt PH and those with borderline PH was detected when they focused on patients with Group 1 PH [7]. In our study, the patients in Group 3 had a poor prognosis, even in the non-PH group. Further, the number of non-PH patients was higher during 1999–2009 than that during 2010–2020, since we recently tended to perform RHC only in patients who may benefit from treatment with vasodilators. This means that most non-PH patients in 1999–2009 may not have received better treatment, compared with those diagnosed in 2010–2020. In addition, the number of patients who died due to malignant disease or who underwent lung transplantation was higher in the non-PH group. These underlying conditions may have affected the poor prognosis of the non-PH group in Group 3. Additionally, even when focusing on pre-capillary PH in Group 3, conventional PH vs. borderline PH and age were poor prognostic factors; however, the factors were not statistically significant. These data suggest that hemodynamics alone did not always determine the patients’ prognosis in Group 3. Similarly, the Japanese Group 3 PH registry revealed that in combined pulmonary fibrosis and emphysema, and interstitial pneumonia, the major cause of death was either respiratory failure or progression or acute exacerbation of underlying disease [18]. The higher percentage of patients in Group 3 in our study may also explain why there was no significant difference in survival between the conventional PH and non-PH groups (Fig. 2).

Concerning survival with different PVR cut-offs, Xanthouli et al. recently showed that patients with pre-capillary borderline PH with PVR ≥ 2 WU had a significantly poorer prognosis than those with PVR < 2 WU in patients with systemic sclerosis [3]. Following this study, we compared the survival between patients with borderline pre-capillary PH (25 > mPAP > 20 mmHg and PAWP ≤ 15 mmHg) with PVR ≥ 3WU and those with PVR of 2–3 WU. There was no significant difference in survival between these two definition groups (Fig. 4). Additionally, both patients died due to underlying diseases other than right heart failure. These data indicated that patients with PVR of 2–3 WU may not always have a good prognosis, which corresponds to the findings of Xanthouli et al. regarding patients with scleroderma. Further studies are needed to confirm whether this new definition is beneficial in clinical practice, including the cut-off for PVR.

Furthermore, the follow-up data for borderline PH and borderline pre-capillary PH was small, and very few patients were administered vasodilators during the follow-up (Table 8). The poor prognosis and unknown cause of death in these patients warrants the necessity for a closer follow-up to detect the progression of PH. Accordingly, prospective studies are needed to evaluate whether the new definition is valuable in identifying patients with PH and those who require prescription of vasodilators.

## Limitation

This was a retrospective single-center study, and the sample size was relatively small to evaluate the pre-capillary PH group effectively. Furthermore, the possibility of selection bias could not be discounted, since our PH center specializes in respiratory medicine and the incidence of PH due to heat failure in this study was low. Further, we could not examine extensive lung disease by computed tomography in Group 3.

## Conclusions

This is the first study performed in a PH center in an Asian country to reveal the characteristics of patients with pre-capillary PH, according to the Nice 2018 definition. The Nice 2018 definition accounted for 4.3% of the patients with pre-capillary PH, and most of them were in Groups 3 and 4. It was suggested that hemodynamics alone may not determine the patients’ prognosis. Further prospective studies are needed to determine whether this new definition is beneficial in clinical practice and provides relevant information regarding prescription of PH-specific treatment.

## Data Availability

The study database was anonymized, and the study complied with the requirements of the Japanese Ministry of Health, Labour and Welfare. The datasets analyzed during the current study are not publicly available, but are available from the corresponding author on a reasonable request and with the permission of our department.

## Abbreviations

WSPH: The World Symposium on Pulmonary Hypertension
PH: Pulmonary hypertension
mPAP: Mean pulmonary arterial pressure
RHC: Right heart catheterization
CO: Cardiac output
PAWP: Pulmonary arterial wedge pressure
PVR: Pulmonary vascular resistance
PEA: Pulmonary endarterectomy
ANOVA: One-way analysis of variance
AaDO_2_: Alveolar-arterial oxygen difference
PaCO_2_: Partial pressure of arterial carbon dioxide
PaO_2_: Partial pressure of arterial oxygen
PvO_2_: Partial pressure of mixed venous oxygen
WU: Wood unit
